# Arrhythmias among Older Adults Receiving Comprehensive Geriatric Care: Prevalence and Associated Factors

**DOI:** 10.3390/clinpract14010011

**Published:** 2024-01-04

**Authors:** Marco Meyer, Andreas Arnold, Thomas Stein, Ulrich Niemöller, Christian Tanislav, Damir Erkapic

**Affiliations:** 1 Department of Geriatrics, Diakonie Hospital Jung-Stilling Siegen, Wichernstrasse 40, 57074 Siegen, Germany; 2 Department of Cardiology and Rhythmology, Diakonie Hospital Jung-Stilling Siegen, Wichernstrasse 40, 57074 Siegen, Germany; 3 University Hospital, Justus Liebig University Giessen, 35392 Giessen, Germany

**Keywords:** geriatrics, arrhythmia, Holter ECG

## Abstract

Background: Cardiovascular diseases and arrhythmias are medical conditions that increase with age and are associated with significant morbidities and mortality. The aim of the present study was to investigate the prevalence of arrhythmias and clinical associations in the collective of older adults receiving comprehensive geriatric care (CGC). Methods: Holter ECG monitoring (HECG) of older patients hospitalized for CGC was analyzed. The prevalence of arrhythmias and the associations between the presence of arrhythmias, patients’ characteristics and the functional status regarding basic activities of daily living (assessed by the Barthel index (BI)), walking ability (assessed by the timed up and go test (TUG)), and balance and gait (assessed by the Tinetti balance and gait test (TBGT)) were examined. Results: In the presented study, 626 patients were included (mean age: 83.9 ± 6.6 years, 67.7% were female). The most common arrhythmias detected in HECG were premature ventricular contractions (87.2%), premature atrial contractions (71.7%), and atrial fibrillation (22.7%). Atrial flutter was found in 1.0%, paroxysmal supraventricular tachycardia in 5.8%, non-sustained ventricular tachycardia in 12.5%, first-degree AV block in 0.8%, second-degree AV block type Mobitz I in 0.8%, second-degree AV block type Mobitz II in 0.3%, pause > 2.5 s any cause in 3.5%, and pause > 3 s any cause in 1.6% of the cases. Premature atrial contractions were associated with the female sex (74.8% vs. 65.3%, *p* = 0.018), whereas in male patients, the following arrhythmias were more common: premature ventricular contractions (91.6% vs. 85.1%, *p* = 0.029), ventricular bigeminus (8.4% vs. 3.8%, *p* = 0.021), and non-sustained ventricular tachycardia (17.3% vs. 10.1%, *p* = 0.014). Atrial fibrillation detected in HECG was more frequent in patients at high risk of falls, indicated by their TBGT score ≤ 18 (24.7% vs. 12.0%, *p* = 0.006), and premature ventricular contractions were more common in patients unable to walk (TUG score 5) compared to those with largely independent mobility (TUG score 1 or 2) (88.0% vs. 75.0%, *p* = 0.023). In a logistic regression analysis, atrial fibrillation detected in HECG was identified as a risk factor for a high risk of falls (odds ratio (OR): 2.35, 95% confidence interval (CI): 1.23–4.46). Conclusion: In our study, investigation of HECG of older adults hospitalized for CGC revealed that premature atrial contractions, premature ventricular contractions, and atrial fibrillation were the most common arrhythmias. Premature atrial contractions were found to be more frequent in female patients, while male patients were more prone to premature ventricular contractions. In the investigated population, atrial fibrillation emerged as a risk factor associated with a high risk of falls.

## 1. Introduction

Cardiac arrhythmias are a prevalent health issue posing a significant challenge for healthcare systems. Age dependency of arrhythmia is observed in several arrhythmia conditions such as atrial and ventricular premature beats, paroxysmal supraventricular tachycardia, non-sustained ventricular tachycardia, atrioventricular block, or particularly, atrial fibrillation (AF) [1,2]. An investigation conducted by Khurshid et al. including over 500,000 people, revealed that 2.35% exhibited a baseline rhythm abnormality, with prevalence increasing with age. In this population, AF, bradyarrhythmias, and conduction system diseases were more common than supraventricular and ventricular arrhythmias [3]. The presence of cardiac arrhythmia is also relevant in regard to patients’ functional status. In a prospective study examining cardiac arrhythmias and physical performance of older people, it was shown that arrhythmia at baseline was associated with the incidence of disability in activities of daily living and worse performance in balance tests [4]. AF, as one of the most frequent types of arrhythmia, affected approximately 43.6 million people globally in 2016. The lifetime risk of developing AF for individuals of European ancestry at the age of 55 is one in three [5,6,7]. It is projected that the number of people (aged 55 years and over) diagnosed with AF in the European Union will more than double from 8.8 million in 2010 to 17.9 million by 2060 [8]. Epidemiological data confirm the highest prevalence of AF among patients 80 years and older with variation from 10 to 17% [9].

The clinical relevance of arrhythmia, especially AF, is significant and evident in its outcomes and complications. It is estimated that 20–30% of all ischemic strokes are caused by AF, and 20–30% of heart failure patients are diagnosed with this condition. AF is associated with increased mortality, cognitive and physical decline, high hospitalization rates, and reduced quality of life [5,10]. Frailty, as a syndrome that increases with age causes reduced physical capabilities and due to its relationship to AF, is an important component of current research. Frail patients are more prone to new-onset AF and those with pre-existing AF are at increased risk for serious adverse outcome events such as thromboembolic complications (ischemic stroke), bleeding, and all-cause death [11,12].

The high impact of arrhythmia on health care systems can also be demonstrated by the number of pacemaker implantations, with high-degree atrioventricular block (AV block) and sick sinus syndrome as the most common indications. In the year 2009, approximately 225,000 pacemakers were implanted in the United States and the highest number of new implants was documented in Germany (927 per million). Despite national differences, older adults over the age of 60 consistently represent the largest group of pacemaker recipients [1,13]. 

Overall, arrhythmia is a commonly diagnosed and diverse clinical condition in everyday clinical practice, and its prevalence is increasing among older adults. For these reasons, we aimed to investigate the diversity and prevalence of arrhythmias in a special population of hospitalized older people who underwent comprehensive geriatric care (CGC).

## 2. Materials and Methods

### 2.1. Patients

From 1231 patients hospitalized in our clinic for geriatrics between May 2019 and April 2020, 626 patients received Holter ECG monitoring (HECG) during their hospital stay and were selected for this retrospective, single-center analysis. 

### 2.2. Comprehensive Geriatric Care

Comprehensive geriatric care (CGC) encompasses a specialized treatment framework designed to meet the complex needs of older patients. These patients are referred by their primary care physicians, specialists, or other medical facilities. The scope of treatment within our geriatric department is diverse, encompassing individuals with immediate medical conditions, those in post-operative recovery, and those diagnosed with common geriatric syndromes (such as frailty, mobility issues, dizziness, malnutrition, incontinence, and cognitive decline) [14,15,16]. The main objectives of CGC include medical treatment, improvement of functional deficits, and preservation of independence. To achieve these goals, CGC employs a multidisciplinary approach. A thorough assessment is conducted to evaluate mobility, cognitive and emotional capabilities, basic activities of daily living (ADL), and social circumstances. A multiprofessional team, comprising experienced geriatricians, nursing staff, psychologists, social workers, dietitians, and various therapist professions (occupational/physio/speech therapists) collaboratively determine an individualized treatment strategy for each patient. CGC regularly spans a minimum of two weeks, during which a minimum of 20 treatment units (each lasting 30 min) are scheduled. These sessions encompass physiotherapy, occupational therapy, logopedics, or psychological care. The treatment goals for the individual patient are discussed in weekly team conferences and may be adjusted if necessary.

### 2.3. Barthel Index

The Barthel index (BI) is an established assessment tool that serves to evaluate patients’ functional independence concerning basic activities of daily living. These encompass essential areas such as dressing, eating, bathing, toileting, and mobility. The BI scale ranges from 0 to 100, with higher scores indicating greater autonomy and capability for independent execution of these activities [17]. A BI score of 35 indicates severe motor function impairment and was defined as a cut-off value in our study [18,19].

### 2.4. Tinetti Balance and Gait Test

The Tinetti balance and gait test (TBGT), is a standardized clinical assessment tool to evaluate balance and gait function. This assessment consists of two primary components: the balance assessment, where patients perform tasks such as rising from and sitting down on a chair and attempting to rise and turning 360°, and the gait assessment, which involves observing aspects of walking like step pattern and trunk stability. The total score achievable in the TBGT is 28 points. The higher the score, the better the performance [20]. A TBGT score ≤ 18 implicates high risk of falling and was selected as a cut-off value in the presented investigation [21,22].

### 2.5. Timed up and Go Test (TUG)

The timed up and go test (TUG) is a standardized clinical assessment to evaluate patients’ walking ability and potential fall risk. In this test, the patient sits on a chair, stands up, walks three meters, turns around, returns to the chair, and sits down. The time taken to complete the test is measured, with an extended duration indicating compromised mobility or an elevated likelihood of falling. Defined TUG categories: (5) patient not able to walk; (4) >30 s performing TUG; (3) 20–29 s performing the TUG; (2) 10–19 s for TUG completion; (1) <10 s for TUG completion. A TUG score of 1 indicates unrestricted walking ability, patients with a TUG score of 2 tend to be independently mobile, and a TUG score of 3 or 4 implicates mobility restriction relevant to daily life [23].

### 2.6. Holter ECG Monitoring

Holter ECG monitoring (HECG) is a routinely performed diagnostic procedure in our geriatric department. HECG was scheduled for 24 h with wearable ECG devices (custo flash 510 and custo flash 200, custo med GmbH, Ottobrunn, Germany). 

Data sets were stored and evaluated offline within the manufacturer’s provided system (custo diagnostic, custo med GmbH, Ottobrunn, Germany). Examinations were evaluated by a cardiologist in the clinical care routine. For this retrospective study, findings of the routinely performed HECG examinations were evaluated for the presence of the following arrhythmias: premature atrial contractions (PACs), premature ventricular contractions (PVCs), ventricular bigeminus, ventricular couplet and non-sustained ventricular tachycardia (NSVT, defined as run of consecutive ventricular beats persisting for 3 beats to 30 s [24]), paroxysmal supraventricular tachycardia (PSVT), atrial fibrillation (AF), atrial flutter, first to third degree atrioventricular block (AV block), pause > 2.5 s, and pause > 3 s. 

Indication for HECG was based on individual physicians’ judgement. Reviewing the case files, the following disorders/reasons for performing HECG were identified:

- history of falls
- history of syncope
- currently reported dizziness
- currently reported palpitations
- current antiarrhythmic therapy
- suspected paroxysmal atrial fibrillation
- therapy management of atrial fibrillation (rhythm- or rate-controlling therapies)

### 2.7. Data Collection

**Figure 1:**
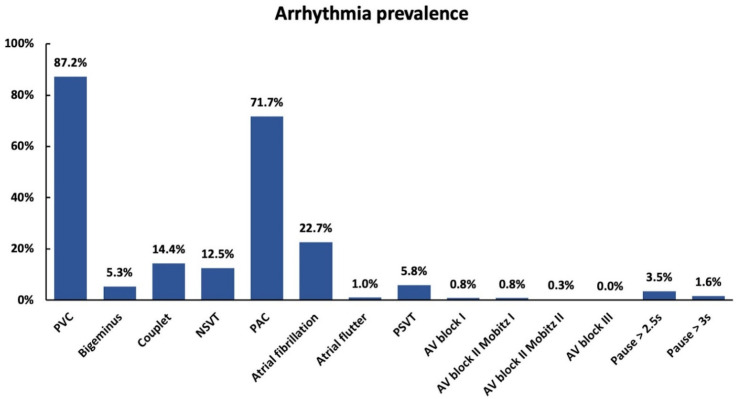
Arrhythmias found in Holter ECG monitoring among older adults hospitalized for CGC. PVC: premature ventricular contraction; PAC: premature atrial contraction; PSVT: paroxysmal supraventricular tachycardia; NSVT: non-sustained ventricular tachycardia; AV block: atrioventricular block.

For the present study, the case files of patients who were treated in our geriatric department between May 2019 and April 2020 and received HECG during their hospital stay were evaluated (*n* = 626). Demographic parameters (age and sex), patients’ comorbidities (listed in Table 1; acute and previous stroke/intracranial hemorrhage, intracranial tumor, and unspecified head injuries were summarized as structural brain lesion), HECG findings (illustrated in Figure 1), and scores in the geriatric assessment (BI, TBGT, and TUG) on hospital admission were documented for further statistical analysis. 

### 2.8. Statistical Analyses

Demographic parameters, comorbidities, and HECG findings were documented in the clinical care routine and evaluated for this retrospective analysis. Data sets were presented as mean ± standard deviation (SD), median, and interquartile range (IQR), as well as percentages and frequencies. Fisher’s exact test for categorical data was carried out. Normal distribution was checked by the Kolmogorov–Smirnov test. Analysis of unpaired samples was carried out using the Mann–Whitney U-test for non-normally distributed data and the t-test in case of normal distribution. Clinical factors associated with high risk of falls indicated by a TBGT score ≤ 18 (with a *p*-value < 0.05 between groups in univariate analysis) were entered into logistic regression analysis. Statistical analysis was performed using PSPP software (version 1.4.1, GNU project).

### 2.9. Ethical Approval 

We received ethical approval for this retrospective study (Ethical committee of the Medical Chamber Westfalen-Lippe and of the Westfälische Wilhelms-Universität Münster (University of Münster, Germany) protocol number: 2021-175-f-S).

## 3. Results

In the present study, 626 patients were included (mean age: 83.9 ± 6.6 years, 67.7% were female). Specifically, cardiovascular comorbidities such as hypertension was diagnosed in 83.9%, diabetes mellitus in 31.5%, heart failure in 24.8%, coronary heart disease in 32.4%, and AF in 38.5% of the cases. A cardiac pacemaker was present in 8.1%. BI on hospital admission was median 45 (IQR: 30–60), TUG median 4 (IQR: 3–5), and TBGT median 11 (IQR: 2–16). Patients’ characteristics and comorbidities are presented in Table 1.

Considering patients’ characteristics by sex, female patients were older (84.4 ± 6.6 vs. 82.9 ± 6.5, *p* = 0.007). Diabetes mellitus (38.6% vs. 28.1%, *p* = 0.010), coronary heart disease (45.0% vs. 26.4%, *p* < 0.001), AF (46.5% vs. 34.7%, *p* = 0.005), and structural brain lesion (34.7% vs. 21.5%, *p* = 0.001) were more frequent in the male sex, whereas current fracture was associated with the female sex (55.9% vs. 30.7%, *p* < 0.001). Scores in functional assessments did not differ significantly by sex (BI in female patients median 45 (IQR: 35–55) vs. BI in male patients median 45 (IQR: 25–60), *p* = 0.748; TUG in female patients median 4 (IQR: 3–5) vs. TUG in male patients median 4 (IQR: 3–5), *p* = 0.272; TBGT in female patients median 11 (IQR: 2–16) vs. TBGT in male patients median 11 (IQR: 3–17), *p* = 0.274).

Patients with BI ≤ 35 compared to those with BI > 35 were older (84.9 ± 6.7 vs. 83.3 ± 6.5, *p* = 0.004). Dementia (32.5% vs. 15.1%, *p* < 0.001) and structural brain lesion (35.1% vs. 20.4%, *p* < 0.001) were more common in patients with BI ≤ 35. Depression was diagnosed more frequently in patients with BI > 35 (15.1% vs. 9.2%, *p* = 0.036) (Table 2). 

Patients with TUG 5 were older than patients with TUG 3 or 4 and TUG 1 or 2 (age: TUG 5 vs. TUG 1 or 2: 84.7 ± 6.7 vs. 82.2 ± 6.9, *p* = 0.018; TUG 5 vs. TUG 3 or 4: 84.7 ± 6.7 vs. 83.4 ± 6.3, *p* = 0.012). The frequency of structural brain lesion (TUG 5 vs. TUG 3 or 4: 31.7% vs. 20.8%, *p* = 0.003) and current fracture (TUG 5 vs. TUG 1 or 2: 55.6% vs. 39.6%, *p* = 0.043; TUG 5 vs. TUG 3 or 4: 55.6% vs. 41.7%, *p* = 0.001) was higher in patients with TUG 5. Depression (16.0% vs. 10.2%, *p* = 0.047) was diagnosed more frequently in patients with TUG 3 or 4 compared to the group with TUG 5 (Table 3).

Among those patients with TBGT ≤ 18, diabetes mellitus (33.6% vs. 21.0%, *p* = 0.013), dementia (22.5% vs. 13.0%, *p* = 0.032), and structural brain lesion (27.5% vs. 15.0%, *p* = 0.008) were more frequent than in patients with TBGT > 18. Patients with TBGT ≤ 18 were older (84.2 ± 6.6 vs. 82.6 ± 6.8, *p* = 0.029) (Table 4).

Patients’ characteristics by performance in functional assessments are summarized in Table 2, Table 3 and Table 4.

The most common arrhythmia detected in HECG was PVC with 87.2%. Ventricular bigeminus was found in 5.3%, ventricular couplets in 14.4%, and NSVT in 12.5% of the included patients. PACs were observed in 71.7%. AF was found in 22.7%, atrial flutter in 1.0%, and PSVT in 5.8%. Considering HECG in regard to AV block, first-degree AV block was verified in 0.8%, second-degree AV block type Mobitz I in 0.8%, and second-degree AV block type Mobitz II in 0.3%. Third-degree AV block was not detected. The frequency of pause > 2.5 s was 3.5%, and the frequency of pause > 3 s was 1.6% (Figure 1). Pauses > 2.5 s were caused by AV block II type Mobitz II (4.5%), AV block II type Mobitz I (4.5%), AF (77.4%), compensatory pause after PVC (4.5%), and sinoatrial block at night (9.1%). Pauses > 3 s were caused by AV block II type Mobitz II (10.0%), AV block II type Mobitz I (10.0%), AF (70.0%), and sinoatrial block at night (10%).

In female patients, PACs (74.8% vs. 65.3%, *p* = 0.018), and in male patients, PVCs (91.6% vs. 85.1%, *p* = 0.029), ventricular bigeminus (8.4% vs. 3.8%, *p* = 0.021) and NSVT (17.3% vs. 10.1%, *p* = 0.014) were more frequent (Table 5). 

No differences were found in arrhythmia prevalence among patients with BI ≤ 35 compared to those with BI > 35 (Table 2).

PVCs were more common in patients with TUG 5 compared to patients with TUG 1 or 2 (88.0% vs. 75.0%, *p* = 0.023) (Table 3).

AF (24.7% vs. 12.0%, *p* = 0.006) was more common in the HECG of patients with TBGT ≤ 18 (Table 4). 

In a logistic regression analysis, AF detected in HECG (odds ratio (OR): 2.35, 95% confidence interval (CI) 1.23–4.46), and the comorbidities diabetes mellitus (OR: 1.84, 95% CI: 1.09–3.12), dementia (OR: 1.99, 95% CI: 1.06–3.73), and structural brain lesion (OR: 2.02, 95% CI: 1.12–3.65) were identified as risk factors for high risk of falls (indicated by TBGT score ≤ 18) (Table 6).

## 4. Discussion

In the present study, the highest arrhythmia prevalence was documented for ectopic beats with 71.7% for premature atrial contractions and 87.2% for premature ventricular contractions, respectively. Atrial fibrillation as a high-risk factor for disabling diseases such as stroke and heart failure was found in 22.7% of the HECG. 

Arrhythmia is a clinical condition that increases with age based on a variety of pathophysiological processes. Aging is associated with progressive degenerative changes in the contractile and conduction systems. Electrical remodeling, structural changes, autonomic nervous system dysregulation, electrolyte imbalances, oxidative stress, concomitant diseases, and medication side effects disrupt the heart’s normal electrical activity and may contribute to the development of supraventricular and ventricular arrhythmias [1,25,26,27].

Considering frequencies of arrhythmias in our study, PVCs and PACs emerged as the most common arrhythmia patterns in the HECG examinations. This finding aligns with several previous investigations [2,28,29]. The results of the cardiovascular health study by Maniolo et al., investigating arrhythmia in 24 h ambulatory ECG of older adults, revealed that both ventricular and supraventricular ectopic activity are extremely common in older people, with only 18% and 2.8%, respectively, showing no such occurrences [29]. In a separate study focusing on PACs in Japanese men, it was shown that 99% of participants had at least one PAC during 1 h. The authors found a significant increase in the number of PACs with age, with the median number of PACs per hour rising from 1.04 in patients aged 40–59 to 4.19 in those aged 70–79 years old [2]. Conen et al. investigated PACs in the general population and reached similar conclusions on PAC frequency and age distribution [30].

Regarding PVCs, a high prevalence has also been supported by a series of examinations [28,31,32,33]. In a recent study from Dong et al., which focused on outpatients with palpitations, the prevalence of PVCs was found to be 67.7%, increasing with age and reaching 84.6% in individuals aged 80 years and older. In the study population of Dong and colleagues, a significantly higher PVC prevalence was observed in men, with the greatest sex-specific differences seen in individuals over 60 years of age [28]. These findings are consistent with the results of our examination, that also found a higher frequency of PVCs in men. Overall, data on sex distribution of PVCs are not clear. In contrast to our findings and those of Dong et al., a population-based cohort study found a higher age-adjusted incidence of symptomatic PVCs in females [34]. The reasons for these sex-specific frequency differences are speculative. It could be discussed that the study populations are quite different. Sirichand and colleagues investigated a cohort with an average age of 52.1 years, while our study population had a mean age of 83.9 years, more than 1.5 times higher, and had higher rates of cardiovascular risk factors [34]. In regard to patients’ functionality, performance of less physical activity was associated with greater PVC frequency, as indicated by the investigation of Marcus et al. [35]. This assertion is further substantiated by the findings of our investigation, which revealed that PVCs were particularly prevalent in patients unable to walk compared to those with largely unrestricted mobility. In the present study, the risk factors diabetes mellitus and coronary heart disease, as arrhythmia-favoring conditions, were significantly more common in men than in women. Overall, the clinical relevance of ectopic beats can be discussed controversially, since it is also a very common phenomenon in the general population. However, few studies have shown that PVCs have prognostic relevance in regard to mortality of people with and without heart disease [31,32].

Considering NSVT, an extensive investigation by Lin et al. revealed its prognostic significance in relation to various outcomes, including death, cardiovascular-related hospitalization, ischemic stroke, and new-onset heart failure. In this population, patients with NSVT were older and predominantly male; diabetes mellitus and hypertension diagnoses were associated with NSVT occurrence. The findings of Lin et al. support the results of the present study, which also identified that males are particularly affected by NSVT [36].

In our investigation, AF is the second most common arrhythmia after ectopic beats and was observed in 22.7% of the HECG. AF poses a significant challenge for healthcare systems in the 21st century, given its escalating prevalence and potential for severe associated conditions, such as stroke and heart failure. In a cross-sectional examination conducted in Italy among subjects aged 65 and older, the prevalence of AF was estimated to be approximately 1.08 million in 2016, and it is projected to increase by 75% to about 1.89 million people by 2060. Similar predictions have been made by the authors for the European Union, with an estimated 89% increase in AF prevalence from 2016 to 2060. In 2016, 53.5% of individuals diagnosed with AF in the Italian study population were aged at least 80 years, and it is projected that this percentage will rise to 69.6% in that age group by 2060. In the European Union, AF prevalence is predicted to increase from 51.2% to 65.2% during the same time period among older adults 80 years and above [37]. Previous data indicate a higher prevalence of AF in men [38,39]. When examining sex differences in cardiovascular comorbidities within our study population, it becomes evident that the diagnosis of AF is also more frequent in men. However, this sex discrepancy is not significantly pronounced in the HECG findings. The rate of detectable AF during the inpatient stay, as recorded in the HECG, was notably lower than the proportion of patients diagnosed with AF (22.7% versus 38.5%). This point refers, on the one hand, to the potential lack of detectability in cases of paroxysmal AF during HECG, and on the other hand, to the effects of rhythm-controlling measures. 

AF is not only clinically significant due to its high prevalence, but it also holds considerable prognostic importance. The presence of AF impacts both mortality and morbidity in affected individuals, leads to restrictions of the functional status, and furthermore impacts quality of life [5]. The impact of AF on quality of life and exercise tolerance is evident in more than 60% of AF patients, with women, younger individuals, and those with comorbidities particularly affected [5,40,41,42,43]. AF prevalence in relation to patients’ functionality is of particular relevance. Several studies revealed the association between AF and functional impairment. Frailty as a syndrome of reduced physical capabilities and enhanced vulnerability is a common phenomenon among older adults diagnosed with AF. On the one hand, frail patients are at higher risk for new-onset AF and on the other hand, frail patients already diagnosed with AF are at higher risk for all-cause death, ischemic stroke, and bleeding [11,12]. In addition to that, frail patients were less likely to be treated with oral anticoagulants, which might increase the risk of further thromboembolic complications [44]. Considering patients’ functional status, we found that AF detected in HECG during patients’ hospital stay was associated with a high risk of falls, indicated by TBGT score ≤ 18. Supporting our results, Koca and colleagues documented higher fall rates among AF patients in their study [10]. These results align with the research conducted by Hung et al. who also demonstrated that AF diagnosis is associated with falls in the older population [45]. Furthermore, disability in basic and instrumental activities of daily living and slower walking speed was observed in patients with an AF diagnosis [10,46]. In an examination by Donoghue et al. focusing on community-dwelling older adults, associations between AF and mobility were investigated. The study revealed that AF was independently linked to lower usual gait speed, and this effect was more pronounced in participants aged 70 years and older [47].

The frequency of other cardiac arrhythmias, which were found in addition to AF and ectopic beats in HECG, was much lower. Nevertheless, the occurrence of these arrhythmias is not less relevant in clinical practice. Thus, for example, higher-grade AV block is particularly significant for those affected, as these cardiac arrhythmias may require the use of a pacemaker device [1]. Higher-grade AV block second-degree type Mobitz II was found in 0.3% of the HECG, and third-degree AV block was not detected. For comparison, a Chinese study with a very large sample size of 15 million participants revealed prevalence rates of 0.42‰ for second-degree AV block, and 0.1‰ for third-degree AV block in participants aged 60 years and older [48].

As with further arrhythmias like AF, the occurrence of AV block was associated with age and cardiac risk factors such as hypertension and diabetes mellitus, respectively [48,49,50]. Contrary to the findings of other studies, our research did not reveal a sex-specific association with a more frequent occurrence of AV block in males [48,49,50].

In summary, our study offers an overview of the prevalence of various cardiac arrhythmias in older adults, providing valuable insights into this age group’s cardiac health.

The major strength of this study is its substantial sample size, consisting of 626 hospitalized geriatric patients, with a notably high mean age of 83.9 years. Furthermore, the study benefits from the inclusion of a diverse treatment spectrum offered in the geriatric clinic, encompassing a wide range of surgical and medical diagnoses. This allows for a comprehensive analysis of a broad patient population. However, limitations should also be taken into account. A major limitation of the present study is the absence of a control group. To address this limitation and provide valuable insights into the epidemiology of arrhythmia in older people, future research projects could consider comparing the prevalence of arrhythmia between older adults requiring inpatient treatment and community-dwelling, healthy individuals within the same age range. A selection bias could be considered as a further limitation of the study is that it was an individual geriatrician’s decision which patients were selected for CGC and underwent HECG.

## 5. Conclusions

In our study investigating HECG of older adults hospitalized for CGC, premature atrial contractions, premature ventricular contractions, and atrial fibrillation were the most common arrhythmias. Premature atrial contractions were found to be more frequent in female patients, while male patients were more prone to premature ventricular contractions. In the investigated population, atrial fibrillation emerged as a risk factor associated with a high risk of falls.

## Figures and Tables

**Table 1:** Patients’ characteristics.

	(*n* = 626)
Age (mean ± SD, years)	83.9 ± 6.6
Sex	
Female	424 (67.7%)
Male	202 (32.3%)
Comorbidities	
Hypertension	525 (83.9%)
Diabetes mellitus	197 (31.5%)
Heart failure	155 (24.8%)
Coronary heart disease	203 (32.4%)
Atrial fibrillation	241 (38.5%)
Cardiac pacemaker	51 (8.1%)
Carcinoma/Tumor	130 (20.8%)
Chronic obstructive pulmonary disease	66 (10.5%)
Asthma	16 (2.6%)
Dementia	134 (21.4%)
Depression	81 (12.9%)
Structural brain lesion ^#^	161 (25.7%)
Current fracture	299 (47.8%)
Fracture of the thorax, ribs, sternum	13 (2.1%)
Fracture of the cranium	9 (1.4%)
Fracture of the pelvis	43 (6.9%)
Fracture of the spinal column	81 (12.9%)
Fracture of the upper extremities	34 (5.4%)
Fracture of the lower extremities	148 (23.6%)
Functional assessments	
Barthel index on admission (*n* = 626) *	45 (30–60) 44.54 ± 19.24
Timed up and go test on admission (*n* = 620) *	4 (3–5) 4.07 ± 1.01
Tinetti test on admission (*n* = 606) *	11 (2–16) 10.30 ± 7.62

^#^ includes acute and previous stroke/intracranial hemorrhage, intracranial tumor and unspecified head injuries. * presented as median and interquartile range (IQR) and mean ± standard deviation (SD).

**Table 2:** Comorbidities and Holter ECG findings by Barthel index (BI). BI ≤ 35 indicates severe motor impairment.

	Total Group	BI ≤ 35	BI > 35	*p*-Value
	(*n* = 626)	(*n* = 228)	(*n* = 398)	
Age (mean ± SD, years)	83.9 ± 6.6	84.9 ± 6.7	83.3 ± 6.5	0.004
Sex				
Female	424 (67.7%)	149 (65.4%)	275 (69.1%)	0.374
Male	202 (32.3%)	79 (34.6%)	123 (30.9%)	
Comorbidities				
Hypertension	525 (83.9%)	189 (82.9%)	336 (84.4%)	0.652
Diabetes mellitus	197 (31.5%)	79 (34.6%)	118 (29.6%)	0.211
Heart failure	155 (24.8%)	54 (23.7%)	101 (25.4%)	0.700
Coronary heart disease	203 (32.4%)	66 (28.9%)	137 (34.4%)	0.183
Atrial fibrillation	241 (38.5%)	91 (39.9%)	150 (37.7%)	0.609
Cardiac pacemaker	51 (8.1%)	15 (6.6%)	36 (9.0%)	0.293
Carcinoma/tumor	130 (20.8%)	44 (19.3%)	86 (21.6%)	0.540
Chronic obstructive pulmonary disease	66 (10.5%)	27 (11.8%)	39 (9.8%)	0.421
Asthma	16 (2.6%)	4 (1.8%)	12 (3.0%)	0.435
Dementia	134 (21.4%)	74 (32.5%)	60 (15.1%)	<0.001
Depression	81 (12.9%)	21 (9.2%)	60 (15.1%)	0.036
Structural brain lesion ^#^	161 (25.7%)	80 (35.1%)	81 (20.4%)	<0.001
Current fracture	299 (47.8%)	119 (52.2%)	180 (45.2%)	0.097
Arrhythmias detected in Holter ECG				
Atrial fibrillation	142 (22.7%)	55 (24.1%)	87 (21.9%)	0.552
Atrial flutter	6 (1.0%)	3 (1.3%)	3 (0.8%)	0.673
Paroxysmal supraventricular tachycardia (PSVT)	36 (5.8%)	16 (7.0%)	20 (5.0%)	0.372
Non-sustained ventricular tachycardia (NSVT)	78 (12.5%)	35 (15.4%)	43 (10.8%)	0.103
Premature atrial contractions (PACs)	449 (71.7%)	160 (70.2%)	289 (72.6%)	0.520
Premature ventricular contractions (PVCs)	546 (87.2%)	204 (89.5%)	342 (85.9%)	0.216
Bigeminus	33 (5.3%)	16 (7.0%)	17 (4.3%)	0.142
Couplet	90 (14.4%)	37 (16.2%)	53 (13.3%)	0.344
AV block I	5 (0.8%)	4 (1.8%)	1 (0.3%)	0.062
AV block II Type Mobitz I	5 (0.8%)	1 (0.4%)	4 (1.0%)	0.658
AV block II Type Mobitz II	2 (0.3%)	1 (0.4%)	1 (0.3%)	>0.999
AV block III	0 (0.0%)	0 (0.0%)	0 (0.0%)	
Pause > 2.5 s any cause	22 (3.5%)	10 (4.4%)	12 (3.0%)	0.375
Pause > 3 s any cause	10 (1.6%)	5 (2.2%)	5 (1.3%)	0.509
Functional assessments				
Timed up and go test on admission (*n* = 620) *	4 (3–5)	5 (5–5)	4 (3–5)	<0.001
	4.07 ± 1.01	4.77 ± 0.54	3.67 ± 1.00	
Tinetti test on admission (*n* = 606) *	11 (2–16)	2 (0–6.5)	15 (10–18.5)	<0.001
	10.30 ± 7.62	4.07 ± 5.29	13.77 ± 6.41	

^#^ includes acute and previous stroke/intracranial hemorrhage, intracranial tumor, and unspecified head injuries. * presented as median and interquartile range (IQR) and mean ± standard deviation (SD).

**Table 3:** Comorbidities and Holter ECG findings by patients’ score in timed up and go test (TUG). Patients unable to walk vs. patients with largely unrestricted walking ability (TUG 5 vs. TUG 1 or 2) and vs. those with mobility restrictions (TUG 5 vs. TUG 3 or 4).

	Total Group	TUG 5	TUG	*p*-Value	TUG	*p*-Value
			1 or 2		3 or 4	
	(*n* = 620)	(*n* = 284)	(*n* = 48)		(*n* = 288)	
Age (mean ± SD, years)	83.9 ± 6.6	84.7 ± 6.7	82.2 ± 6.9	0.018	83.4 ± 6.3	0.012
Sex						
Female	421 (67.9%)	199 (70.1%)	29 (60.4%)	0.183	193 (67.0%)	0.472
Male	199 (32.1%)	85 (29.9%)	19 (39.6%)		95 (33.0%)	
Comorbidities						
Hypertension	520 (83.9%)	236 (83.1%)	37 (77.1%)	0.311	247 (85.8%)	0.420
Diabetes mellitus	195 (31.5%)	92 (32.4%)	9 (18.8%)	0.063	94 (32.6%)	>0.999
Heart failure	154 (24.8%)	68 (23.9%)	9 (18.8%)	0.579	77 (26.7%)	0.501
Coronary heart disease	200 (32.3%)	88 (31.0%)	14 (29.2%)	0.867	98 (34.0%)	0.475
Atrial fibrillation	238 (38.4%)	114 (40.1%)	19 (39.6%)	>0.999	105 (36.5%)	0.390
Cardiac pacemaker	51 (8.2%)	24 (8.5%)	4 (8.3%)	>0.999	23 (8.0%)	0.880
Carcinoma/tumor	129 (20.8%)	55 (19.4%)	12 (25.0%)	0.436	62 (21.5%)	0.536
Chronic obstructive pulmonary disease	66 (10.6%)	32 (11.3%)	4 (8.3%)	0.801	30 (10.4%)	0.789
Asthma	16 (2.6%)	6 (2.1%)	1 (2.1%)	>0.999	9 (3.1%)	0.602
Dementia	131 (21.1%)	70 (24.6%)	8 (16.7%)	0.272	53 (18.4%)	0.083
Depression	80 (12.9%)	29 (10.2%)	5 (10.4%)	>0.999	46 (16.0%)	0.047
Structural brain lesion ^#^	159 (25.6%)	90 (31.7%)	9 (18.8%)	0.087	60 (20.8%)	0.003
Current fracture	297 (47.9%)	158 (55.6%)	19 (39.6%)	0.043	120 (41.7%)	0.001
Arrhythmias detected in Holter ECG						
Atrial fibrillation	141 (22.7%)	68 (23.9%)	7 (14.6%)	0.192	66 (22.9%)	0.844
Atrial flutter	6 (1.0%)	3 (1.1%)	0 (0.0%)	>0.999	3 (1.0%)	>0.999
Paroxysmal supraventricular tachycardia (PSVT)	35 (5.6%)	19 (6.7%)	1 (2.1%)	0.330	15 (5.2%)	0.484
Non-sustained ventricular tachycardia (NSVT)	77 (12.4%)	31 (10.9%)	6 (12.5%)	0.804	40 (13.9%)	0.311
Premature atrial contractions (PACs)	444 (71.6%)	199 (70.1%)	37 (77.1%)	0.391	208 (72.2%)	0.581
Premature ventricular contractions (PVCs)	540 (87.1%)	250 (88.0%)	36 (75.0%)	0.023	254 (88.2%)	>0.999
Bigeminus	32 (5.2%)	15 (5.3%)	2 (4.2%)	>0.999	15 (5.2%)	>0.999
Couplet	88 (14.2%)	36 (12.7%)	3 (6.3%)	0.329	49 (17.0%)	0.159
AV block I	5 (0.8%)	4 (1.4%)	0 (0.0%)	>0.999	1 (0.3%)	0.214
AV block II Type Mobitz I	5 (0.8%)	4 (1.4%)	1 (2.1%)	0.544	0 (0.0%)	0.060
AV block II Type Mobitz II	2 (0.3%)	1 (0.4%)	0 (0.0%)	>0.999	1 (0.3%)	>0.999
AV block III	0 (0.0%)	0 (0.0%)	0 (0.0%)		0 (0.0%)	
Pause > 2.5 s any cause	22 (3.5%)	12 (4.2%)	1 (2.1%)	0.701	9 (3.1%)	0.513
Pause > 3 s any cause	10 (1.6%)	6 (2.1%)	1 (2.1%)	>0.999	3 (1.0%)	0.337
Functional assessments						
Barthel index on admission (*n* = 620) *	45 (30–60)	30 (20–40)	70 (60–80)	<0.001	55 (45–65)	<0.001
	44.64 ± 19.26	31.53 ± 13.52	68.33 ± 14.99		53.61 ± 15.44	
Tinetti test on admission (*n* = 604) *	11 (2–16)	2 (0–5)	21.5 (20–24)	<0.001	15 (12–18)	<0.001
	10.30 ± 7.63	3.38 ± 3.87	21.73 ± 3.15		14.87 ± 4.38	

^#^ includes acute and previous stroke/intracranial hemorrhage, intracranial tumor, and unspecified head injuries. * presented as median and interquartile range (IQR) and mean ± standard deviation (SD).

**Table 4:** Comorbidities and Holter ECG findings by Tinetti balance and gait test (TBGT). TBGT ≤ 18 indicates high risk of falling.

	Total Group	TBGT	TBGT	*p*-Value
		≤18	>18	
	(*n* = 606)	(*n* = 506)	(*n* = 100)	
Age (mean ± SD, years)	83.9 ± 6.6	84.2 ± 6.6	82.6 ± 6.8	0.029
Sex				
Female	411 (67.8%)	346 (68.4%)	65 (65.0%)	0.558
Male	195 (32.2%)	160 (31.6%)	35 (35.0%)	
Comorbidities				
Hypertension	506 (83.5%)	425 (84.0%)	81 (81.0%)	0.463
Diabetes mellitus	191 (31.5%)	170 (33.6%)	21 (21.0%)	0.013
Heart failure	150 (24.8%)	126 (24.9%)	24 (24.0%)	0.900
Coronary heart disease	193 (31.8%)	163 (32.2%)	30 (30.0%)	0.725
Atrial fibrillation	230 (38.0%)	201 (39.7%)	29 (29.0%)	0.055
Cardiac pacemaker	48 (7.9%)	39 (7.7%)	9 (9.0%)	0.685
Carcinoma/tumor	126 (20.8%)	100 (19.8%)	26 (26.0%)	0.177
Chronic obstructive pulmonary disease	65 (10.7%)	53 (10.5%)	12 (12.0%)	0.600
Asthma	15 (2.5%)	14 (2.8%)	1 (1.0%)	0.485
Dementia	127 (21.0%)	114 (22.5%)	13 (13.0%)	0.032
Depression	74 (12.2%)	62 (12.3%)	12 (12.0%)	>0.999
Structural brain lesion ^#^	154 (25.4%)	139 (27.5%)	15 (15.0%)	0.008
Current fracture	290 (47.9%)	245 (48.4%)	45 (45.0%)	0.584
Arrhythmias detected in Holter ECG				
Atrial fibrillation	137 (22.6%)	125 (24.7%)	12 (12.0%)	0.006
Atrial flutter	6 (1.0%)	6 (1.2%)	0 (0.0%)	0.596
Paroxysmal supraventricular tachycardia (PSVT)	34 (5.6%)	32 (6.3%)	2 (2.0%)	0.098
Non-sustained ventricular tachycardia (NSVT)	72 (11.9%)	55 (10.9%)	17 (17.0%)	0.091
Premature atrial contractions (PACs)	434 (71.6%)	354 (70.0%)	80 (80.0%)	0.052
Premature ventricular contractions (PVCs)	528 (87.1%)	446 (88.1%)	82 (82.0%)	0.103
Bigeminus	31 (5.1%)	25 (4.9%)	6 (6.0%)	0.622
Couplet	89 (14.7%)	78 (15.4%)	11 (11.0%)	0.283
AV block I	5 (0.8%)	5 (1.0%)	0 (0.0%)	>0.999
AV block II Type Mobitz I	5 (0.8%)	5 (1.0%)	0 (0.0%)	>0.999
AV block II Type Mobitz II	2 (0.3%)	2 (0.4%)	0 (0.0%)	>0.999
AV block III	0 (0.0%)	0 (0.0%)	0 (0.0%)	
Pause > 2.5 s any cause	21 (3.5%)	20 (4.0%)	1 (1.0%)	0.228
Pause > 3 s any cause	9 (1.5%)	8 (1.6%)	1 (1.0%)	>0.999
Functional assessments				
Barthel index on admission (*n* = 606) *	45 (30–60)	40 (30–50)	67.5 (55–80)	<0.001
	45.06 ± 19.11	40.90 ± 17.13	66.10 ± 14.12	
Timed up and go test on admission (*n* = 604) *	4 (3–5)	5 (4–5)	3 (2–3)	<0.001
	4.04 ± 1.01	4.34 ± 0.80	2.57 ± 0.62	

^#^ includes acute and previous stroke/intracranial hemorrhage, intracranial tumor, and unspecified head injuries. * presented as median and interquartile range (IQR) and mean ± standard deviation (SD).

**Table 5:** Comorbidities and Holter ECG findings by sex.

	Total Group	Female	Male	*p*-Value
	(*n* = 626)	(*n* = 424)	(*n* = 202)	
Age (mean ± SD, years)	83.9 ± 6.6	84.4 ± 6.6	82.9 ± 6.5	0.007
Comorbidities				
Hypertension	525 (83.9%)	361 (85.1%)	164 (81.2%)	0.245
Diabetes mellitus	197 (31.5%)	119 (28.1%)	78 (38.6%)	0.010
Heart failure	155 (24.8%)	106 (25.0%)	49 (24.3%)	0.921
Coronary heart disease	203 (32.4%)	112 (26.4%)	91 (45.0%)	<0.001
Atrial fibrillation	241 (38.5%)	147 (34.7%)	94 (46.5%)	0.005
Cardiac pacemaker	51 (8.1%)	28 (6.6%)	23 (11.4%)	0.060
Carcinoma/tumor	130 (20.8%)	64 (15.1%)	66 (32.7%)	<0.001
Chronic obstructive pulmonary disease	66 (10.5%)	27 (6.4%)	39 (19.3%)	<0.001
Asthma	16 (2.6%)	11 (2.6%)	5 (2.5%)	>0.999
Dementia	134 (21.4%)	88 (20.8%)	46 (22.8%)	0.603
Depression	81 (12.9%)	62 (14.6%)	19 (9.4%)	0.075
Structural brain lesion ^#^	161 (25.7%)	91 (21.5%)	70 (34.7%)	0.001
Current fracture	299 (47.8%)	237 (55.9%)	62 (30.7%)	<0.001
Arrhythmias detected in Holter ECG				
Atrial fibrillation	142 (22.7%)	90 (21.2%)	52 (25.7%)	0.221
Atrial flutter	6 (1.0%)	4 (0.9%)	2 (1.0%)	>0.999
Paroxysmal supraventricular tachycardia (PSVT)	36 (5.8%)	21 (5.0%)	15 (7.4%)	0.270
Non-sustained ventricular tachycardia (NSVT)	78 (12.5%)	43 (10.1%)	35 (17.3%)	0.014
Premature atrial contractions (PACs)	449 (71.7%)	317 (74.8%)	132 (65.3%)	0.018
Premature ventricular contractions (PVCs)	546 (87.2%)	361 (85.1%)	185 (91.6%)	0.029
Bigeminus	33 (5.3%)	16 (3.8%)	17 (8.4%)	0.021
Couplet	90 (14.4%)	55 (13.0%)	35 (17.3%)	0.180
AV block I	5 (0.8%)	3 (0.7%)	2 (1.0%)	0.660
AV block II Type Mobitz I	5 (0.8%)	3 (0.7%)	2 (1.0%)	0.660
AV block II Type Mobitz II	2 (0.3%)	1 (0.2%)	1 (0.5%)	0.542
AV block III	0 (0.0%)	0 (0.0%)	0 (0.0%)	
Pause > 2.5 s any cause	22 (3.5%)	12 (2.8%)	10 (5.0%)	0.244
Pause > 3 s any cause	10 (1.6%)	6 (1.4%)	4 (2.0%)	0.734
Functional assessments				
Barthel index on admission (*n* = 626) *	45 (30–60)	45 (35–55)	45 (25–60)	0.748
	44.54 ± 19.24	44.27 ± 18.28	45.12 ± 21.13	
Timed up and go test on admission (*n* = 620) *	4 (3–5)	4 (3–5)	4 (3–5)	0.272
	4.07 ± 1.01	4.1 ± 1.0	4.0 ± 1.03	
Tinetti test on admission (*n* = 606) *	11 (2–16)	11 (2–16)	11 (3–17)	0.274
	10.30 ± 7.62	10.09 ± 7.63	10.74 ± 7.58	

^#^ includes acute and previous stroke/intracranial hemorrhage, intracranial tumor, and unspecified head injuries. * presented as median and interquartile range (IQR) and mean ± standard deviation (SD).

**Table 6:** Logistic regression analysis: clinical factors associated with high risk of falls (indicated by Tinetti balance and gait test (TBGT) score ≤18).

Variable	B	S.E.	Wald	*p*-Value	OR (95% CI)
Atrial fibrillation (in HECG)	0.85	0.33	6.78	0.009	2.35 (1.23–4.46)
Diabetes mellitus	0.61	0.27	5.19	0.023	1.84 (1.09–3.12)
Dementia	0.69	0.32	4.61	0.032	1.99 (1.06–3.73)
Structural brain lesion ^#^	0.70	0.30	5.43	0.020	2.02 (1.12–3.65)

^#^ includes acute and previous stroke/intracranial hemorrhage, intracranial tumor, and unspecified head injuries. B: regression coefficient; S.E.: standard error; OR: odds ratio; 95% CI: 95% confidence interval.

## Data Availability

Data supporting the findings of the presented study are available upon reasonable request from the corresponding author.

## References

[1] Chow G.V., Marine J.E., Fleg J.L. (2012). Epidemiology of arrhythmias and conduction disorders in older adults. Clin. Geriatr. Med..

[2] Ahmed S., Hisamatsu T., Kadota A., Fujiyoshi A., Segawa H., Torii S., Takashima N., Kondo K., Nakagawa Y., Ueshima H. (2022). Premature Atrial Contractions and Their Determinants in a General Population of Japanese Men. Circ. J..

[3] Khurshid S., Choi S.H., Weng L.-C., Wang E.Y., Trinquart L., Benjamin E.J., Ellinor P.T., Lubitz S.A. (2018). Frequency of Cardiac Rhythm Abnormalities in a Half Million Adults. Circ. Arrhythm. Eletrophysiol..

[4] Noale M., Veronese N., Smith L., Ungar A., Fumagalli S., Maggi S., Italian Longitudinal Study on Aging Working Group (2020). Associations between cardiac arrhythmia, incident disability in activities of daily living and physical performance: The ILSA study. J. Geriatr. Cardiol..

[5] Hindricks G., Potpara T., Dagres N., Arbelo E., Bax J.J., Blomström-Lundqvist C., Boriani G., Castella M., Dan G.-A., Dilaveris P.E. (2021). 2020 ESC Guidelines for the diagnosis and management of atrial fibrillation developed in collaboration with the European Association for Cardio-Thoracic Surgery (EACTS): The Task Force for the diagnosis and management of atrial fibrillation of the European Society of Cardiology (ESC) Developed with the special contribution of the European Heart Rhythm Association (EHRA) of the ESC. Eur. Heart J..

[6] Staerk L., Wang B., Preis S.R., Larson M.G., Lubitz S.A., Ellinor P.T., McManus D.D., Ko D., Weng L.-C., Lunetta K.L. (2018). Lifetime risk of atrial fibrillation according to optimal, borderline, or elevated levels of risk factors: Cohort study based on longitudinal data from the Framingham Heart Study. BMJ.

[7] Magnussen C., Niiranen T.J., Ojeda F.M., Gianfagna F., Blankenberg S., Njølstad I., Vartiainen E., Sans S., Pasterkamp G., Hughes M. (2017). Sex Differences and Similarities in Atrial Fibrillation Epidemiology, Risk Factors, and Mortality in Community Cohorts: Results From the BiomarCaRE Consortium. Circulation.

[8] Krijthe B.P., Kunst A., Benjamin E.J., Lip G.Y.H., Franco O.H., Hofman A., Witteman J.C.M., Stricker B.H., Heeringa J. (2013). Projections on the number of individuals with atrial fibrillation in the European Union, from 2000 to 2060. Eur. Heart J..

[9] Zoni-Berisso M., Lercari F., Carazza T., Domenicucci S. (2014). Epidemiology of atrial fibrillation: European perspective. Clin. Epidemiol..

[10] Koca M., Yavuz B.B., Tuna Doğrul R., Çalışkan H., Şengül Ayçiçek G., Özsürekçi C., Balcı C., Eşme M., Ünsal P., Halil M. (2020). Impact of atrial fibrillation on frailty and functionality in older adults. Ir. J. Med. Sci..

[11] Hang F., Chen J., Wang Z., Yan J., Wu Y. (2022). Association Between the Frailty and New-Onset Atrial Fibrillation/Flutter Among Elderly Hypertensive Patients. Front. Cardiovasc. Med..

[12] Proietti M., Romiti G.F., Raparelli V., Diemberger I., Boriani G., Dalla Vecchia L.A., Bellelli G., Marzetti E., Lip G.Y., Cesari M. (2022). Frailty prevalence and impact on outcomes in patients with atrial fibrillation: A systematic review and meta-analysis of 1,187,000 patients. Ageing Res. Rev..

[13] Mond H.G., Proclemer A. (2011). The 11th world survey of cardiac pacing and implantable cardioverter-defibrillators: Calendar year 2009-a World Society of Arrhythmia’s project. Pacing Clin. Electrophysiol..

[14] Bell S.P., Vasilevskis E.E., Saraf A.A., Jacobsen J.M.L., Kripalani S., Mixon A.S., Schnelle J.F., Simmons S.F. (2016). Geriatric Syndromes in Hospitalized Older Adults Discharged to Skilled Nursing Facilities. J. Am. Geriatr. Soc..

[15] Inouye S.K., Studenski S., Tinetti M.E., Kuchel G.A. (2007). Geriatric Syndromes: Clinical, Research, and Policy Implications of a Core Geriatric Concept. J. Am. Geriatr. Soc..

[16] Möller J., Rausch C., Laflamme L., Liang Y. (2022). Geriatric syndromes and subsequent health-care utilization among older community dwellers in Stockholm. Eur. J. Ageing..

[17] Mahoney F.I., Barthel D.W. (1965). Functional Evaluation: The Barthel Index. Md. State Med. J..

[18] Bundesinstitut für Arzneimittel und Medizinprodukte BfArM (Federal Institute for Drugs and Medical Devices of Germany) (2023). ICD-10-GM Version 2023, ICD-Code: U50.40 [Internet]. https://www.dimdi.de/static/de/klassifikationen/icd/icd-10-gm/kode-suche/htmlgm2023/block-u50-u52.htm.

[19] gesund.bund.de. A Service from the Federal Ministry of Health (Germany) (2023). U50.40: Severe Motor Function Impairment: Barthel Index Score: 20-35 [Internet]. https://gesund.bund.de/en/icd-code-search/u50-40.

[20] Tinetti M.E. (1986). Performance-oriented assessment of mobility problems in elderly patients. J. Am. Geriatr. Soc..

[21] Köpke S., Meyer G. (2006). The Tinetti test: Babylon in geriatric assessment. Z. Gerontol. Geriatr..

[22] Curcio F., Basile C., Liguori I., Della-Morte D., Gargiulo G., Galizia G., Testa G., Langellotto A., Cacciatore F., Bonaduce D. (2016). Tinetti mobility test is related to muscle mass and strength in non-institutionalized elderly people. Age.

[23] Podsiadlo D., Richardson S. (1991). The timed “Up & Go”: A test of basic functional mobility for frail elderly persons. J. Am. Geriatr. Soc..

[24] Zeppenfeld K., Tfelt-Hansen J., de Riva M., Winkel B.G., Behr E.R., Blom N.A., Charron P., Corrado D., Dagres N., de Chillou C. (2022). 2022 ESC Guidelines for the management of patients with ventricular arrhythmias and the prevention of sudden cardiac death. Eur. Heart J..

[25] Curtis A.B., Karki R., Hattoum A., Sharma U.C. (2018). Arrhythmias in Patients ≥80 Years of Age: Pathophysiology, Management, and Outcomes. J. Am. Coll. Cardiol..

[26] Karagueuzian H.S., Nguyen T.P., Qu Z., Weiss J.N. (2013). Oxidative stress, fibrosis, and early afterdepolarization-mediated cardiac arrhythmias. Front. Physiol..

[27] North B.J., Sinclair D.A. (2012). The intersection between aging and cardiovascular disease. Circ. Res..

[28] Dong Y., Li X., Zheng W., Man Y., Liu J., Yu P., Zhang F., Yang B., Cao K. (2022). Prevalence and heart rate variability characteristics of premature ventricular contractions detected by 24-hour Holter among outpatients with palpitations in China: A cross-sectional study. BMJ Open.

[29] Manolio T.A., Furberg C.D., Rautaharju P.M., Siscovick D., Newman A.B., Borhani N.O., Gardin J.M., Tabatznik B. (1994). Cardiac arrhythmias on 24-h ambulatory electrocardiography in older women and men: The Cardiovascular Health Study. J. Am. Coll. Cardiol..

[30] Conen D., Adam M., Roche F., Barthelemy J.-C., Felber Dietrich D., Imboden M., Künzli N., von Eckardstein A., Regenass S., Hornemann T. (2012). Premature atrial contractions in the general population: Frequency and risk factors. Circulation.

[31] Ahn M.-S. (2013). Current Concepts of Premature Ventricular Contractions. J. Lifestyle Med..

[32] Hirose H., Ishikawa S., Gotoh T., Kabutoya T., Kayaba K., Kajii E. (2010). Cardiac mortality of premature ventricular complexes in healthy people in Japan. J. Cardiol..

[33] Ng G.A. (2006). Treating patients with ventricular ectopic beats. Heart.

[34] Sirichand S., Killu A.M., Padmanabhan D., Hodge D.O., Chamberlain A.M., Brady P.A., Kapa S., Noseworthy P.A., Packer D.L., Munger T.M. (2017). Incidence of Idiopathic Ventricular Arrhythmias: A Population-Based Study. Circ. Arrhythm. Electrophysiol..

[35] Marcus G.M. (2020). Evaluation and Management of Premature Ventricular Complexes. Circulation.

[36] Lin C.-Y., Chang S.-L., Chung F.-P., Chen Y.-Y., Lin Y.-J., Lo L.-W., Hu Y.-F., Tuan T.-C., Chao T.-F., Liao J.-N. (2016). Long-Term Outcome of Non-Sustained Ventricular Tachycardia in Structurally Normal Hearts. PLoS ONE.

[37] Di Carlo A., Bellino L., Consoli D., Mori F., Zaninelli A., Baldereschi M., Cattarinussi A., D’Alfonso M.G., Gradia C., Sgherzi B. (2019). Prevalence of atrial fibrillation in the Italian elderly population and projections from 2020 to 2060 for Italy and the European Union: The FAI Project. Europace.

[38] Andrade J., Khairy P., Dobrev D., Nattel S. (2014). The clinical profile and pathophysiology of atrial fibrillation: Relationships among clinical features, epidemiology, and mechanisms. Circ. Res..

[39] Kavousi M. (2020). Differences in Epidemiology and Risk Factors for Atrial Fibrillation between Women and Men. Front. Cardiovasc. Med..

[40] Freeman J.V., Simon D.N., Go A.S., Spertus J., Fonarow G.C., Gersh B.J., Hylek E.M., Kowey P.R., Mahaffey K.W., Thomas L.E. (2015). Association Between Atrial Fibrillation Symptoms, Quality of Life, and Patient Outcomes: Results From the Outcomes Registry for Better Informed Treatment of Atrial Fibrillation (ORBIT-AF). Circ. Cardiovasc. Qual. Outcomes.

[41] Blum S., Muff C., Aeschbacher S., Ammann P., Erne P., Moschovitis G., Di Valentino M., Shah D., Schläpfer J., Fischer A. (2017). Prospective Assessment of Sex-Related Differences in Symptom Status and Health Perception Among Patients with Atrial Fibrillation. J. Am. Heart Assoc..

[42] Walters T.E., Wick K., Tan G., Mearns M., Joseph S.A., Morton J.B., Sanders P., Bryant C., Kistler P.M., Kalman J.M. (2019). Symptom severity and quality of life in patients with atrial fibrillation: Psychological function outweighs clinical predictors. Int. J. Cardiol..

[43] Randolph T.C., Simon D.N., Thomas L., Allen L.A., Fonarow G.C., Gersh B.J., Kowey P.R., Reiffel J.A., Naccarelli G.V., Chan P.S. (2016). Patient factors associated with quality of life in atrial fibrillation. Am. Heart J..

[44] Proietti M., Romiti G.F., Vitolo M., Harrison S.L., Lane D.A., Fauchier L., Marin F., Näbauer M., Potpara T.S., Dan G.-A. (2022). Epidemiology and impact of frailty in patients with atrial fibrillation in Europe. Age Ageing.

[45] Hung C.-Y., Wu T.-J., Wang K.-Y., Huang J.-L., Loh E.-W., Chen Y.-M., Lin C.-S., Lin C.-H., Chen D.-Y., Tan Y.-J. (2013). Falls and Atrial Fibrillation in Elderly Patients. Acta Cardiol. Sin..

[46] Pulignano G., Del Sindaco D., Tinti M.D., Di Lenarda A., Alunni G., Senni M., Tarantini L., Cioffi G., Barbati G., Minardi G. (2016). Atrial fibrillation, cognitive impairment, frailty and disability in older heart failure patients. J. Cardiovasc. Med..

[47] Donoghue O.A., Jansen S., Dooley C., De Rooij S., Van Der Velde N., Kenny R.A. (2014). Atrial Fibrillation Is Associated With Impaired Mobility in Community-Dwelling Older Adults. J. Am. Med. Dir. Assoc..

[48] Shan R., Ning Y., Ma Y., Liu S., Wu J., Fan X., Lv J., Wang B., Li S., Li L. (2021). Prevalence and risk factors of atrioventricular block among 15 million Chinese health examination participants in 2018: A nation-wide cross-sectional study. BMC Cardiovasc. Disord..

[49] Du Z., Xing L., Lin M., Tian Y., Jing L., Yan H., Zhang B., Liu S., Yu S., Sun Y. (2019). Prevalence of first-degree atrioventricular block and the associated risk factors: A cross-sectional study in rural Northeast China. BMC Cardiovasc. Disord..

[50] Kerola T., Eranti A., Aro A.L., Haukilahti M.A., Holkeri A., Junttila M.J., Kenttä T.V., Rissanen H., Vittinghoff E., Knekt P. (2019). Risk Factors Associated With Atrioventricular Block. JAMA Netw. Open.

